# Electroactive Bacteria Associated With Stainless Steel Ennoblement in Seawater

**DOI:** 10.3389/fmicb.2019.00170

**Published:** 2019-02-07

**Authors:** Florian Trigodet, Nicolas Larché, Hilary G. Morrison, Mohamed Jebbar, Dominique Thierry, Loïs Maignien

**Affiliations:** ^1^Univ Brest (UBO), IFREMER, CNRS, Laboratoire de Microbiologie des Environnements Extrêmes, Plouzané, France; ^2^French Corrosion Institute, Brest, France; ^3^Marine Biological Laboratory, Josephine Bay Paul Center for Comparative Molecular Biology and Evolution, Woods Hole, MA, United States

**Keywords:** ennoblement, electroactive bacteria, stainless steel, microbial ecology, 16S rRNA gene

## Abstract

Microorganisms can increase the open-circuit potential of stainless steel immersed in seawater of several hundred millivolts in a phenomenon called ennoblement. It raises the chance of corrosion as the open-circuit potential may go over the pitting corrosion potential. Despite the large impact of the ennoblement, no unifying mechanisms have been described as responsible for the phenomenon. Here we show that the strict electrotroph bacterium “*Candidatus* Tenderia electrophaga” is detected as an ennoblement biomarker and is only present at temperatures at which we observe ennoblement. This bacterium was previously enriched in biocathode systems. Our results suggest that “*Candidatus* Tenderia electrophaga,” and its previously described extracellular electron transfer metabolism coupled to oxygen reduction activity, could play a central role in modulating stainless steel open-circuit potential and consequently mediating ennoblement.

## Introduction

When immersed in oxic seawater, metals and alloys can form an electrochemical cell with metal oxidation as an anode reaction and oxygen reduction at the cathode. In the case of stainless steel, metal (Cr, Ni, Mo,…) oxides form a so called passive layer largely preventing electron flow between these two electrodes. As a consequence, stainless steel exhibit a measurable electrochemical potential between these two half cells, called Open circuit potential (OCP) since no current is drawn from the system. Stainless steel OCP results from the concentration of the reactants, formal half-cell reaction potential (E°’) and the kinetic parameters associated with each half-cell reaction. It can be measured *in situ* using a reference electrode of a known potential. Stainless steel ennoblement is a well-known phenomenon corresponding to an increase of the OCP, typically by 400–500 mV, when these alloys are immersed in seawater (Mollica and Trevis, 1976). As the OCP value gets closer to the pitting corrosion potential, the probability of stainless steel pitting and crevice corrosion initiation increases, hence the central problem raised by ennoblement (Mollica, 1992; Zhang and Dexter, 1995). Ennoblement is a biotic process as it is dependent on microbial colonization and development on the stainless steel surface (Motoda et al., 1990; Scotto and Lai, 1998; Le Bozec et al., 2001; Wei et al., 2005; Gümpel et al., 2006).

Stainless steels are commonly used building material in seawater systems and despite the large industrial impact of the ennoblement, relatively little is known about the diversity and the actual activity of marine microorganisms that colonize stainless steel. There are various hypotheses regarding their possible contributions to ennoblement, as summarized in Little et al. (2008). There are three ways of increasing the OCP: (1) thermodynamics, (2) kinetics and (3) alteration of the nature of the reduction reaction. A decrease of the surface pH could thermodynamically increase the observed OCP. However, as shown by Dexter and Chandrasekaran (2000), the pH changes in a heterogeneous biofilm are highly variable and also challenging to measure. Kinetically, an increase of the cathodic reaction rate can also result in an increase of the OCP. Previous works have demonstrated that surface microorganisms increase cathodic reduction efficiency (Johnsen and Bardal, 1985; Holthe et al., 1989; Audouard et al., 1994; Zhang and Dexter, 1995; Mollica and Scotto, 1996; Rogne and Steinsmo, 1996; Le Bozec et al., 2001; Larché et al., 2011; Thierry et al., 2015). Other reactions such as manganese oxide reduction in freshwater (Dickinson and Lewandowski, 1996; Gümpel et al., 2006) and the formation of hydrogen peroxide could also contribute to the increase of the OCP as it is a stronger oxidant than oxygen with a higher redox potential (Landoulsi et al., 2008b).

While the mechanisms of ennoblement are still discussed, the seawater temperature has been identified as a critical parameter. Ennoblement is a temperature dependent process, undergoing a complete inhibition above a critical temperature around 40°C in temperate seawater and freshwater (Scotto et al., 1986; Dupont et al., 1997; Martin et al., 2003; Gümpel et al., 2006; Thierry et al., 2015). This critical temperature seems to vary with geography as it is 32°C in the Norwegian and Baltic sea (Bardal et al., 1993; Mattila et al., 2000). Despite sustained research on this important potential modulation, no mechanisms have been described that can explain the potential ennoblement, nor could the primary source of electrons for these cathodic reactions be identified.

Some microorganisms can perform direct extracellular electron transfer to and from electrodes. This was demonstrated in controlled systems such as microbial fuel cells. A wide diversity of microorganisms can channel electrons resulting from soluble substrate oxidation toward an anode, thus creating a measurable current in these bioelectrochemical systems (Philips et al., 2015). Far fewer microorganisms were identified as cathodic electron acceptor (Rabaey et al., 2008). Electroactive microorganisms such as *Geobacter sulfurreducens* or *Shewanella oneidensis* have been used as models to understand the electrogenic metabolism or the electron pathway from a soluble electron donor to an anode. However, they were also described as electrotroph, performing the reverse reaction using an electrode as electron donor. *Shewanella oneidensis* is able to switch from an electrogenic metabolism to an electrotrophic by reversing its electron transport pathway (Ross et al., 2011). Similarly, Geobacter species are also able to act as electrotrophs to reduce fumarate to succinate or nitrate to nitrite with electrons provided from a cathode through a direct electron uptake mechanism (Gregory et al., 2004; Strycharz et al., 2011). *Geobacter sulfurreducens* can increase the open-circuit potential by several hundred mV on stainless steel under anoxic condition via an electrotrophic metabolism to reduce fumarate to succinate (Mehanna et al., 2009, 2010). Despite the absence of a model electrotrophic bacterium cultivated under aerobic conditions, some pure cultures were able to catalyze the electrochemical reduction of oxygen (Rabaey et al., 2008; Erable et al., 2010), as well as some environmentally enriched communities (Strycharz-Glaven et al., 2013; Rothballer et al., 2015; Milner et al., 2016; Rimboud et al., 2017). Recently, the study of a bacterial community developed on a biocathode led to the identification of “*Candidatus* Tenderia electrophaga”: a strict electroautotrophic bacterium able to use a cathode as an electron donor to reduce oxygen and able to fix carbon dioxide (Eddie et al., 2016). The presence of electrotrophic bacteria under natural conditions with aerated seawater has not yet been proposed as a possible reason for potential ennoblement despite their apparent ability to change the OCP.

As the rationale for this study, we hypothesize that electrotrophic bacteria could be involved in ennoblement by drawing electrons from immersed stainless steel in open-circuit condition (without additional current provided). However, to our knowledge, no study of stainless steel surface microbial community structure with high throughput sequencing methods has been carried out yet, even less so in relation to modulation of the electrochemical potential of this material. To test our hypothesis, we thus used the temperature dependence property of ennoblement and examine distinctive taxa in ennoblement vs. non ennoblement conditions.

## Materials and Methods

### Materials

The material used in all experiments was super duplex stainless steel (S32750) plates of 100 mm × 50 mm × 10 mm (French Corrosion Institute, France). The nominal composition of the stainless steel is 25.1% Cr, 7.0% Ni, 3.8% Mo, 0.13% Cu, 0.29% N, completed with Fe. The pitting resistance equivalent numbers is 42.3 (%Cr + 3,3%(Mo + 0.5%W) + 16%N). Prior to exposure, the plates were washed for 20 min in 20% nitric acid and sterilized by autoclaving for 20 min at 121°C (dry cycle).

### Experimental Set-Up

We exposed all coupons in 300 L seawater tanks renewed at an approximated rate of 12 L/h with an incoming seawater from the bay of Brest (France) (48°21′32.1″N 4°33′07.4″W).

Seawater tanks were heated at 30, 33, 36, 38, and 40°C (+/-0.5°C). Since only three tanks were available, the experiment was run twice: 30, 33, and 36; and 30, 38, and 40°C one week after the first series. Samples were collected after 7 days of exposure during March 2015.

We also used two natural seawater samples (5 L, *n* = 3) collected from a Bay of Brest coastal microbial observatory close to the tanks’ seawater pump intake. The two samplings took place on the March 02, 2015 and March 26, 2015, before and after the coupon exposure. Seawater was pre-filtered on a 3 μm filter and bacteria collected on a 0.22 μm sterivex filter.

### OCP Measurements and Cathodic Polarization Curves

Stainless steel samples were held by a titanium wire to measure the open-circuit potential with an Ag/AgCl reference electrode. The electrodes were calibrated with saturated calomel electrode (SCE) REF421 (Radiometer, France). The use of titanium wires has been documented in previous works and inhibits galvanic corrosion at the point of contact with stainless steel (Espelid, 2003). Measurements of OCP and temperature were recorded every 30 min. Five replicates were used per condition.

The cathodic polarization curves were drawn on samples exposed for two weeks under the two OCP conditions of interest: with ennoblement at 36°C and without the shift of potential at 40°C. We used a Gamry Reference 600 (Gamry Instruments, United States) from 20 mV over the open-circuit potential to -1.2 V vs. Ag/AgCl electrode with a scan rate of 0.167 mV/s. The dynamic polarization curves started at +20 mV in order to get first points of the anodic branch without perturbing the oxide layer before cathodic scan. All potential values were corrected based on the reference electrode calibration with SCE. We obtained an estimation of the passivation current by drawing the intersection of the tangent of the anodic and cathodic branches close to OCP value.

### SEM Imaging

Dedicated coupons (20 mm × 20 mm × 1.5 mm) were fixed for scanning electronic microscopy (SEM) with 2.5% glutaraldehyde seawater for 1 h, then rinsed three times in seawater for 15 min. The dehydration process involved four washes of 15 min in increasing concentrations of ethanol (50, 70, 90, and 100%) followed by similar washes in hexamethyldisilazane (HMDS) and ethanol solution (⅓ HMDS, ½ HMDS, ⅔ HMDS, and 100% HMDS). We observed surface communities with SEM on samples exposed in seawater at 36 and 40°C using a Hitachi SU3500 machine (Hitachi High-Technologies, Germany). To perform cell counting, we imaged ten random areas for each condition at ×1000 magnification using backscattered-electron imaging. Pictures were processed with the ImageJ software for automatic cell detection. After the background removal, images were converted into binary black and white with the default threshold of the software. The particle analysis was used with a minimum area of 0.5 μm^2^ up to 4 μm^2^ for cell detection (Supplementary Figure 3).

### Surface Cell Collection

Surface cells were collected immediately after coupon collection using a sterile cell lifter (Thermo Fisher Scientific, United States) and by gentle and uniform scratching into 100 mL of a Tris Buffered Saline (TBS) solution (50 mM Tris, 150 mM NaCl, pH 7.6) under sterile conditions ensured by a Bunsen burner, keeping the immediate area sterile. TBS solutions were then stored in ice for transport to the molecular lab. TBS solutions were filtered through 0.22 μm GTTP polycarbonate membranes (Merck Millipore, United States) which were then transferred to PowerBiofilm^®^ Bead Tubes from the PowerBiofilm DNA extraction kit (MoBio, United States). Control samples were collected under identical conditions and visualized by scanning electron microscopy to ensure removal of the cells attached to the surface.

### DNA Extraction and Sequencing

The DNA extraction was performed according to the manufacturer’s instructions of the PowerBiofilm DNA extraction kit (MoBio, United States). The V4–V5 region of the 16S rRNA gene was amplified with the 518F and 926R primers fused with Illumina adapters and sample-specific sets of barcodes and indexes (Nelson et al., 2014). PCR products were visualized on agarose gels and purified with AMPure XP (Agencourt, United States) reagent. DNA concentration was assessed with Quant-iT^TM^ PicoGreen^®^ dsDNA (Invitrogen, United States) prior to pooling the PCR products at equimolar concentration. Sequencing using Illumina MiSeq platform was performed at the Josephine Bay Paul Center (Woods Hole, MA, United States). Sequences were deposited to the European Nucleotide Archive under the accession number PRJEB27599^1^.

### Bioinformatics Analysis

The quality filtering was done following Minoche et al. (2011), recommendations, before merging of paired-end reads with Illumina-Utils python scripts on demultiplexed raw reads (Eren et al., 2013). OTU delineation was performed with the Swarm algorithm using the default local linking threshold *d* = 1 (Mahé et al., 2015). The chimera detection and removal were carried out with VSEARCH (Rognes et al., 2016). The Silva NR 132 database (Quast et al., 2013) was used for taxonomic assignment of Swarm representative sequences with Mothur (Schloss, 2009).

We used the Phyloseq R package to calculate alpha diversity indices and the vegan package to compute beta diversity (with Bray-Curtis indices) and non-metric multidimensional scaling (NMDS) ordination. Stacked bar plots were produced with ggplot2 (Wickham, 2009). We used the two conditions “with ennoblement” (30°C to 38°C) and “without ennoblement” (40°C) to perform biomarker detection with LEfSe (Segata et al., 2011).

A fully reproducible workflow is available at https://loimai.github.io/ennoblement_16S/.

## Results

### Potential Ennoblement on Stainless Steel and Cathodic Polarization Curves

**FIGURE 1:**
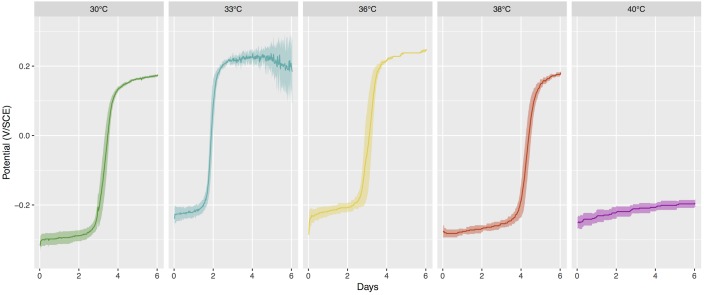
Open-Circuit Potential (OCP) vs. time for stainless steel coupons exposed to different temperature of seawater. Mean value and 95% confidence interval for 5 replicates per conditions, or 10 replicates for 30°C as the two sequential series were pooled together. SCE, saturated calomel electrode.

At the beginning of the incubations, coupons were at - 272 mV ( ± 6 mV) vs. saturated calomel electrode and after 3 to 5 days of incubation, the OCP increased in all coupons except those incubated at 40°C (Figure 1). The potential ennoblement was highly reproducible among replicates at temperatures from 30°C to 38°C with a mean increase of the electrochemical potential of 470 mV ( ± 12 mV) for all samples within 4 days of exposure. In contrast, the open-circuit potential for samples immersed at 40°C changed very little over time (+54 mV, ± 8 mV). In our setup, there was thus a critical temperature between 38°C and 40°C under which ennoblement was observed for all samples with similar maximum electrochemical potential values after day 4 (or day 5 at 38°C), whereas ennoblement did not occur above that temperature.

**FIGURE 2:**
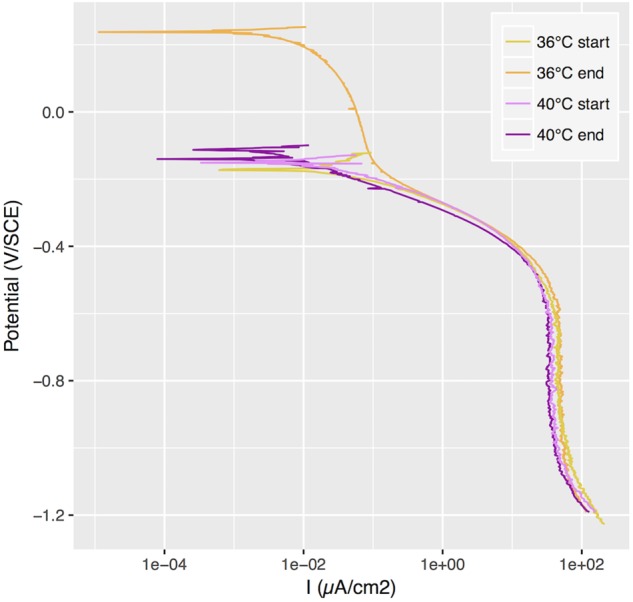
Cathodic polarization curves applied to samples with ennoblement at 36°C and without at 40°C, at the beginning of the exposure and after 2 weeks. SCE, saturated calomel electrode.

In a subsequent incubation under similar conditions, we carried out a cathodic polarization curve on samples exposed at 36°C and 40°C and we observed a shift of the polarization curve after two weeks of exposure at 36°C that was not observed at 40°C (Figure 2). We estimated the passivation current to be around 0.01 μA/cm^2^ for all conditions, based on these polarization curves.

### SEM Observations

**FIGURE 3:**
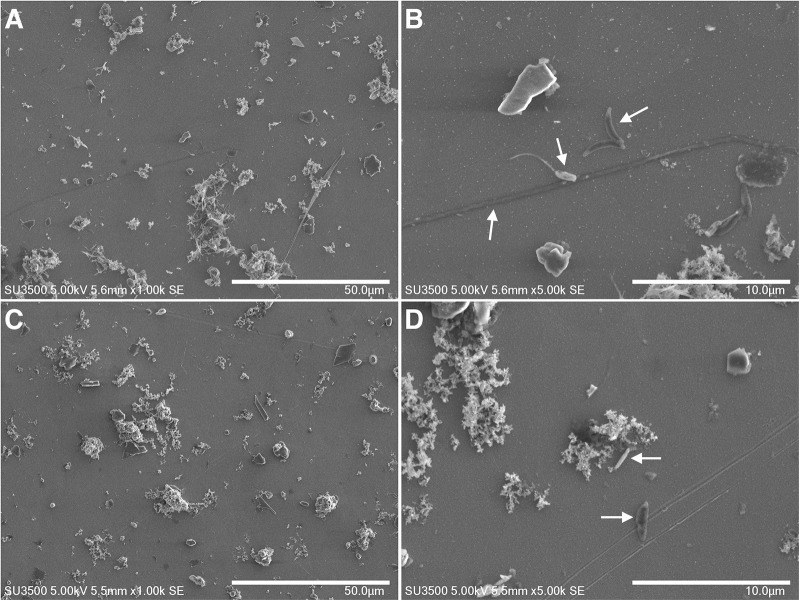
SEM images at 1 k **(A,C)** and 5 k **(B,D)** magnification of stainless steel exposed for one week to seawater and heated to 36°C **(A,B)** and 40°C **(C,D)**. Some cells are pointed by arrows.

We used a similar incubation setup at 36°C and 40°C, allowing biomass colonization on immersed stainless steel coupons for observation of surface communities with SEM. We found an average cell density of 11,661 cells/mm^2^ ( ± 773 cells/mm^2^) at 36°C, and a lower density of 7,219 cells/mm^2^ ( ± 442 cells/mm^2^) at 40°C. Under both conditions, we observed bacilli and coccobacilli, as well as some very long filamentous bacteria, but only at 36°C (see Figure 3).

### Stainless Steel Bacterial Community

We characterized surface bacterial communities for each condition using 16S rRNA amplicon sequencing. We sequenced 36 libraries and obtained 5,597,422 raw sequences of the bacterial 16S rRNA gene V4-V5 region. After quality filtering and paired end read merging, 3,653,018 sequences were retained and clustered into 166,164 operational taxonomic units (OTUs) using the Swarm algorithm with the default local linking threshold *d* = 1 (Mahé et al., 2015). Putative chimeras where removed with the VSEARCH software (Rognes et al., 2016), leading to a high-quality dataset of 66,892 chimera-free OTUs representing 3,240,780 sequences.

**FIGURE 4:**
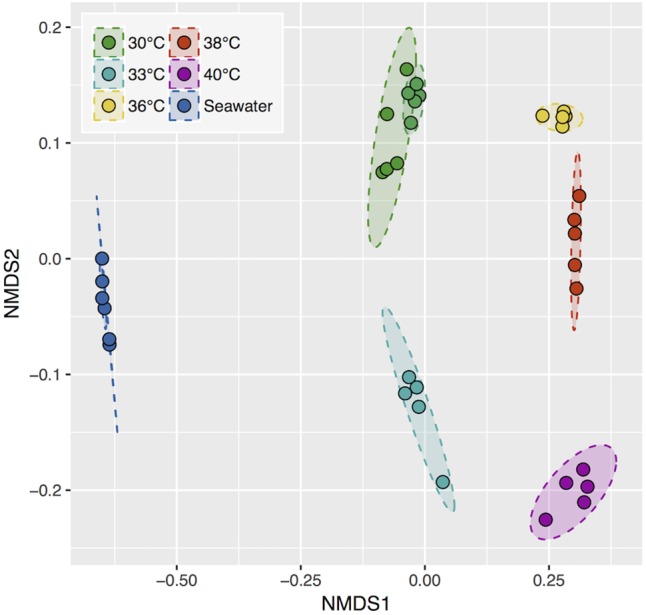
Non-metric multidimensional scaling (NMDS) ordination based on the Bray-Curtis dissimilarity index. 95% confidence area per condition. Stress, 0.086.

Bacterial 16S rRNA diversity in each sample was compared with a weighted dissimilarity index (Bray-Curtis) in an ordination analysis (Figure 4). Replicate samples collected at the same temperature were clustered and differed significantly from one temperature to another. Therefore, the different bacterial communities that develop at temperatures from 30°C up to 38°C appear able to increase the OCP. In addition, the two coupon series incubated at 30°C during a two weeks interval exhibited high community similarity, showing that surface community assembly was highly reproducible.

**FIGURE 5:**
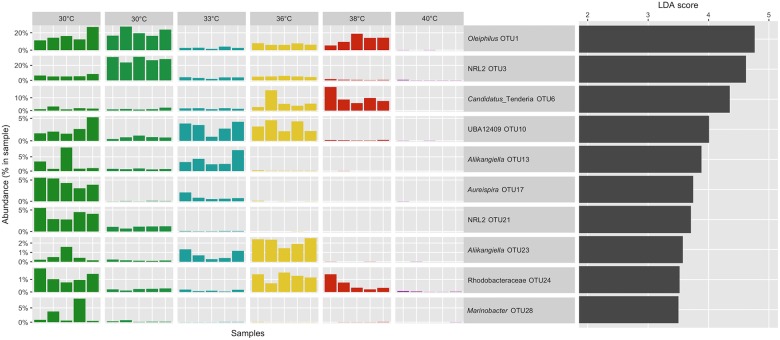
Relative abundance distribution of the ennoblement biomarker OTUs with the 10 best LDA scores. Taxonomic affiliations originate from the Silva 132 database release. Biomarkers were not detected in seawater samples, except for two sequences affiliated to Oleiphilus (OTU1).

We identified microorganisms that were distinctive of the “ennoblement” condition using a biomarker detection analysis with the LEfSe software (Segata et al., 2011). We chose to define the condition with the ennoblement (30, 33, 36, 38°C) as opposed to a lack of OCP change (40°C). We conserved biomarkers with a minimum linear discriminant analysis (LDA) score of 3 resulting in 47 OTUs that were differentially represented during ennoblement. Among these we found mainly Proteobacteria including members of the Oceanospirillales, Rhodobacterales, and Alteromonadales (Figure 5). An OTU affiliated to the genus Oleiphilus genus was remarkably found exclusively in exposures setups between 30°C and 38°C with respective mean relative abundance of 18.41, 2.67, 6.76, 12.40, and 0.03% at 40°C (Figure 5). However, other Oleiphilus OTUs were also present at 40°C. More strikingly, we also detected the presence of a recently described Proteobacteria “*Candidatus* Tenderia electrophaga” as a very strong biomarker (Figure 5). Members of this candidate genus were found in high relative abundance from 30°C to 38°C with mean relative abundance of 1.54, 1.58, 6.64, and 10.05% with a peak abundance of 18.4% in a sample replicate at 38°C (Figure 5). In addition, this bacterium was found exclusively in ennoblement conditions, as no other “*Candidatus* Tenderia electrophaga” OTUs were detected at 40°C.

Finally, we examined the abundance of these biomarker bacteria in the pool of colonizing bacteria from natural seawater collected in the vicinity of our set up pump intake, before and after incubation periods. The bacterial composition in seawater was strikingly different from that of the steel surfaces (Figure 4). No sequences of the best ten biomarker OTUs were recovered from seawater, except for two affiliated to an Oleiphilus OTU.

## Discussion

The open-circuit potential is defined by the concentration of the reactants, formal half-cell potential (E°’) and the kinetic parameters associated with each half-cell reaction. A change in the cathodic reaction has often been invoked as the only half-cell reaction changed by the presence of microorganisms on the surface of the stainless steel. Indeed, the bacterial community is known to increase cathodic reduction efficiency (Johnsen and Bardal, 1985; Holthe et al., 1989; Audouard et al., 1994; Zhang and Dexter, 1995; Mollica and Scotto, 1996; Rogne and Steinsmo, 1996; Le Bozec et al., 2001; Larché et al., 2011). In this study, we were interested in gaining further insight into the bacterial community of the stainless-steel surface immersed in seawater and its electrochemical activity in relation to the potential ennoblement. Previous studies have shown an inhibition of the ennoblement activity above a critical temperature (Scotto et al., 1986; Dupont et al., 1997; Martin et al., 2003; Gümpel et al., 2006; Thierry et al., 2015). In our setting, this critical temperature was between 38°C and 40°C, above which the ennoblement was inhibited despite the continuing presence of bacteria. We used that information to investigate the community composition between ennoblement at lower temperature vs. no ennoblement at higher temperature. A central result of this work is the identification of OTUs affiliated to “*Candidatus* Tenderia electrophaga” that were exclusively present under conditions leading to potential ennoblement, i.e., under 40°C and considerably enriched compared to natural seawater.

Other electroactive bacteria have been shown to be able to change the potential of electrode under anaerobic condition (Mehanna et al., 2009), and a microbial community that was able to do the same in aerobic conditions was described as an electroactive biofilm community (Rimboud et al., 2017). This study is correlative and cannot formally establish a mechanistic link between the detected biomarker and the ennoblement, but the distinctive presence of an electrotroph bacteria in aerobic condition is, to our knowledge, a novel observation and suggest a possible metabolism for potential ennoblement. “*Candidatus* Tenderia electrophaga” can indeed accept electrons from a conductive surface while using oxygen as a terminal electron acceptor (Eddie et al., 2016). This activity is based on its extracellular electron transport system composed of cytochrome c oxidase complexes coupled with the reduction of oxygen and the fixation of carbon dioxide using the Calvin-Benson-Bassham cycle, making it a chemo-electro-autotroph (Eddie et al., 2017). In that study, type IV pili genes were also proposed to play a role in the extracellular electron transport.

In the original study set up that led to the description “*Candidatus* Tenderia electrophaga” on a biocathode, the biofilm development developed a current density between 0.92 and 4.28 μA/cm^2^ at a fixed potential of +66 mV vs. SCE (Malanoski et al., 2018). Our experimental set up does not include biocathodes but rather open-circuit conditions, meaning that no current was provided nor drawn to the surface microbial communities. However, a possible source of electrons could be the passivation current produced by the stainless steel. This current is due to the slow oxidation of iron and chromium atoms in the passive layer of the stainless steel, forming a thin film containing iron and chromium hydroxides (Marcus, 2011). Given the polarization curves obtained at 36 and 40°C we showed that our coupons’ passivation current is on the order of magnitude of 0.01 μA/cm^2^. These values are of two orders of magnitude lower than those observed at microbial fuel cell biocathodes, but could potentially sustain the growth of electroactive bacteria under open-circuit conditions.

Overall, our results suggest that ennoblement could be explained by the following mechanism: the stainless steel would act as an electron source for electrotrophic bacteria via its passivation current, using extracellular electron transport mechanism coupled to oxygen reduction. This hypothesis is based on results of a study using a metabarcoding approach that comes with its own limitations as the sequenced DNA represent a fragment of the 16S rRNA gene and not the complete genome. Also, the biomarker approach does not consider bacteria that could be present at all temperatures but with a different activity at 40°C that would result in the absence of the potential ennoblement. These limitations would be overcome with the use of metagenomics, to assess the genetic potential of the bacterial communities, and metatranscriptomics to confirm if the actual genes expressed would support our hypothesis.

Alternatively, a model for ennoblement based on local pH change at stainless steel surface has been proposed (Dexter and Chandrasekaran, 2000) and requires the formation of a thick biofilm acting as a strong diffusion barrier. Our SEM observations of bacterial colonization after ennoblement do not support this hypothesis, as after one week of exposure, the development of attached bacteria was at a very early stage and could be defined as sparse bacterial colonization rather than an actual biofilm. We did not observe a uniform three-dimensional structure of extracellular polymeric substance with embedded bacteria covering the whole surface of the stainless steel.

Another hypothesis invoked the contribution of hydrogen peroxide as a central electron acceptor during ennoblement (Scotto and Lai, 1998; Landoulsi et al., 2008b). H_2_O_2_ release by heterotrophic bacteria as a byproduct of oxygen respiration, however, requires a high concentration of electron donor (20 mM of D-Glucose in Landoulsi et al., 2008a). This does not correspond to our environmental conditions and is thus unlikely a central explanation for ennoblement in natural seawater environments.

Besides “*Candidatus* Tenderia electrophaga,” other OTUs were identified as biomarker, especially some bacteria able to use aliphatic hydrocarbons as energy and carbon source like Oleiphilus (Yakimov and Golyshin, 2014). These bacteria could originate from the seawater pipes that might be contaminated with a small amount of oil-derived components. This would favor hydrocarbon metabolism and therefore the development of these bacteria. We identified the genus *Marinobacter*, which includes oil degrading species, and which is also found in the cathodic enriched community where “*Candidatus* Tenderia electrophaga” was described by Eddie et al., 2017, and Wang et al., 2015. The presence of oil degrading bacteria in potential ennoblement conditions is intriguing, but their role has yet to be defined. Gammaproteobacteria are often reported in oxygen reducing biocathode communities (Strycharz-Glaven et al., 2013; Rothballer et al., 2015; Milner et al., 2016). They were also found to be dominant in this study (Supplementary Figures 1, 2), and four of the top ten biomarkers are also affiliated to this Gammaproteobacteria (*Oleiphilus*, ‘*Candidatus* Tenderia electrophaga’, *Aliikangiella*, *Marinobacter*). We found other biomarkers with poor taxonomical assignment, only to the order level, e.g., NRL2 (Alphaproteobacteria). Therefore, no hypotheses can be generated from the presence of these biomarkers.

The risk associated with ennoblement is pitting corrosion as the potential increase reach values close to pitting potential. The use of high grade stainless steel and short exposure time limited the risk of pitting corrosion in this study. But future work could involve lower grade stainless steel to associate the bacterial communities to pitting corrosion

## Conclusion

The rational for this study was the observation of a sharp temperature inhibition around 40°C of stainless steel ennoblement in the temperate seawater of the bay of Brest. We used this property to identify bacteria potentially involved in ennoblement. The detection as a biomarker for ennoblement of “*Candidatus* Tenderia electrophaga,” a known electrotroph was a remarkable result. Based on recent literature on “*Candidatus* Tenderia electrophaga” activity, we proposed a new mechanism for ennoblement based on extracellular electron transfer with oxygen as a terminal electron acceptor. The electron donor for this reaction could be the passivation current resulting from slow surface stainless steel oxidation at the passivation layer interface.

## Data Availability Statement

Sequences were deposited to the European Nucleotide Archive under the accession number PRJEB27599 (http://www.ebi.ac.uk/ena/data/view/PRJEB27599).

## Author Contributions

FT, NL, DT, and LM designed and conceived the experiments. FT performed the experiments and data analyses. HM did the sample sequencing. FT and LM drafted the manuscript. NL, DT, HM, and MJ contributed to data interpretation and assisted with writing of the manuscript.

## Conflict of Interest Statement

The authors declare that the research was conducted in the absence of any commercial or financial relationships that could be construed as a potential conflict of interest.

## Footnotes

1 http://www.ebi.ac.uk/ena/data/view/PRJEB27599
