# Supplementary material for: Electroactive Bacteria Associated With Stainless Steel Ennoblement in Seawater
Source: Front Microbiol. 2019 Feb 7;10:170. doi: 10.3389/fmicb.2019.00170 (PMC6374330; doi:10.3389/fmicb.2019.00170)
Supplement: Supplementary file 1 [file Data_Sheet_1.PDF]

Supplementary Material

Electroactive bacteria associated with stainless steel ennoblement in seawater

Florian Trigodet, Nicolas Larché, Hilary G. Morrison, Mohamed Jebbar, Dominique Thierry, Loïs Maignien*

*** Correspondence:** Loïs Maignien: lois.maignien@univ-brest.fr

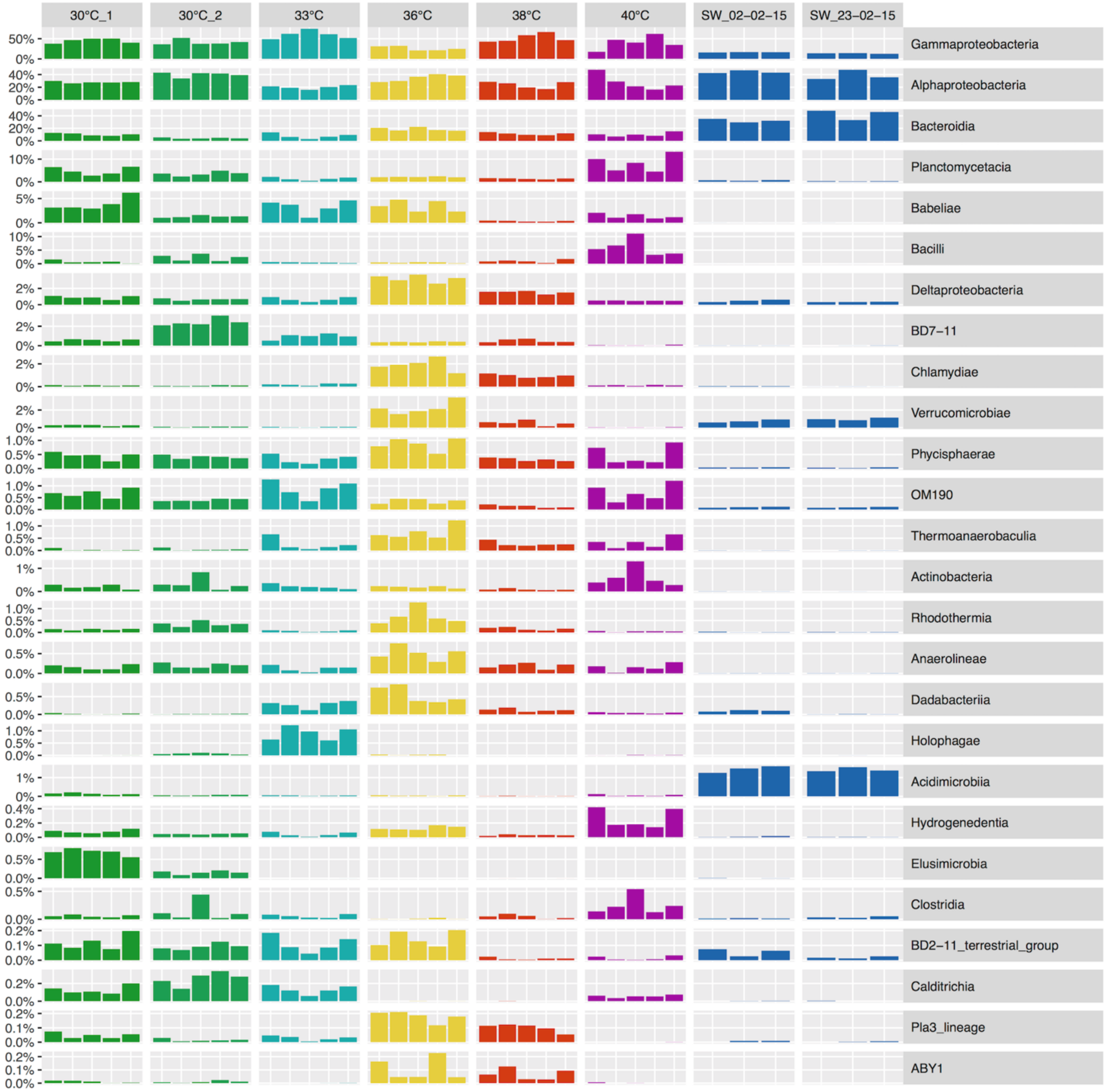

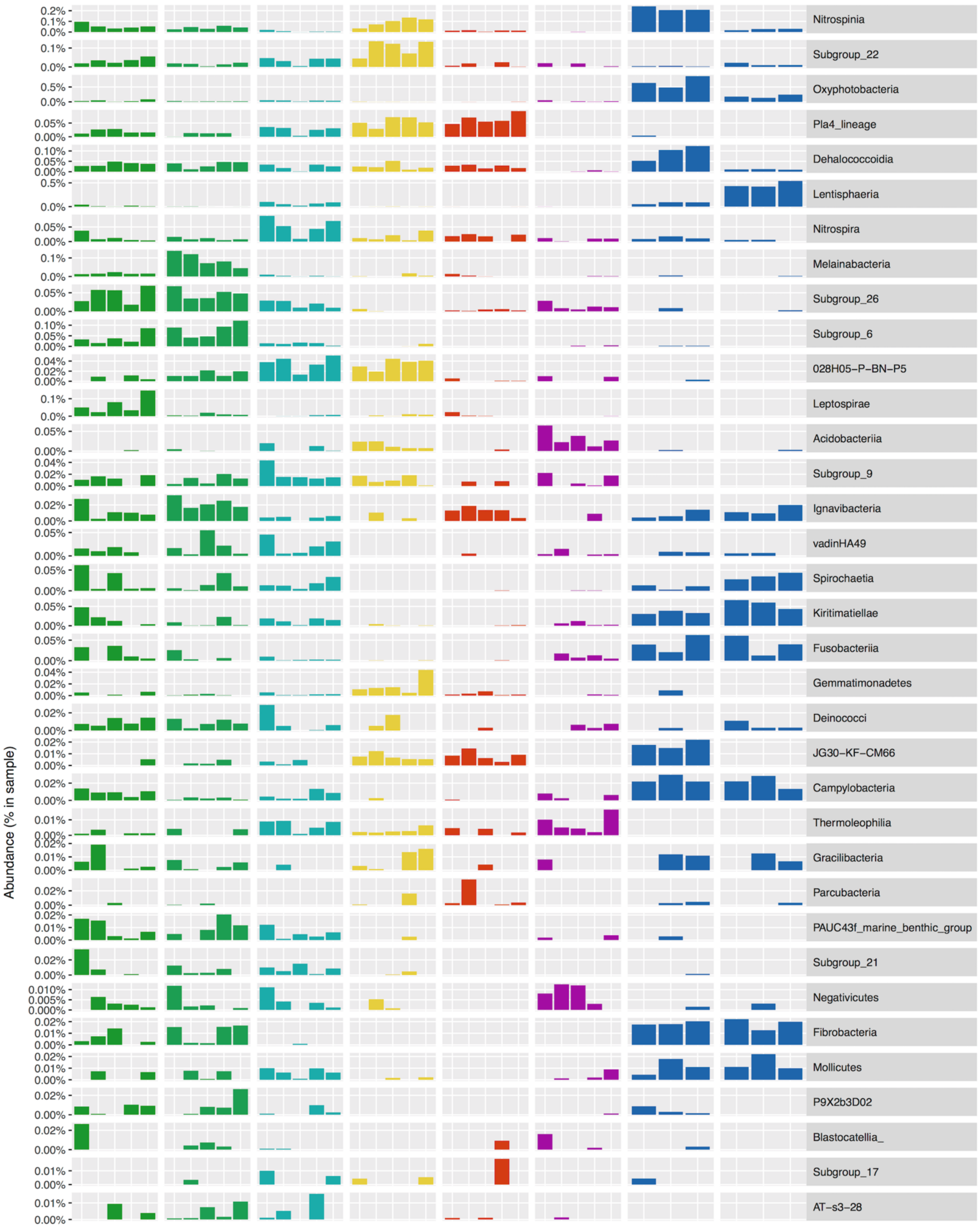
Abundance (% in sample)
Nitrospinia
Subgroup_22
Oxyphotobacteria
Pla4_lineage
Dehalococcoidia
Lentisphaeria
Nitrospira
Melainabacteria
Subgroup_26
Subgroup_6
028H05−P−BN−P5
Leptospirae
Acidobacteriia
Subgroup_9
Ignavibacteria
vadinHA49
Spirochaetia
Kiritimatiellae
Fusobacteriia
Gemmatimonadetes
Deinococci
JG30−KF−CM66
Campylobacteria
Thermoleophilia
Gracilibacteria
Parcubacteria
PAUC43f_marine_benthic_group
Subgroup_21
Negativicutes
Fibrobacteria
Mollicutes
P9X2b3D02
Blastocatellia_
Subgroup_17
AT−s3−28

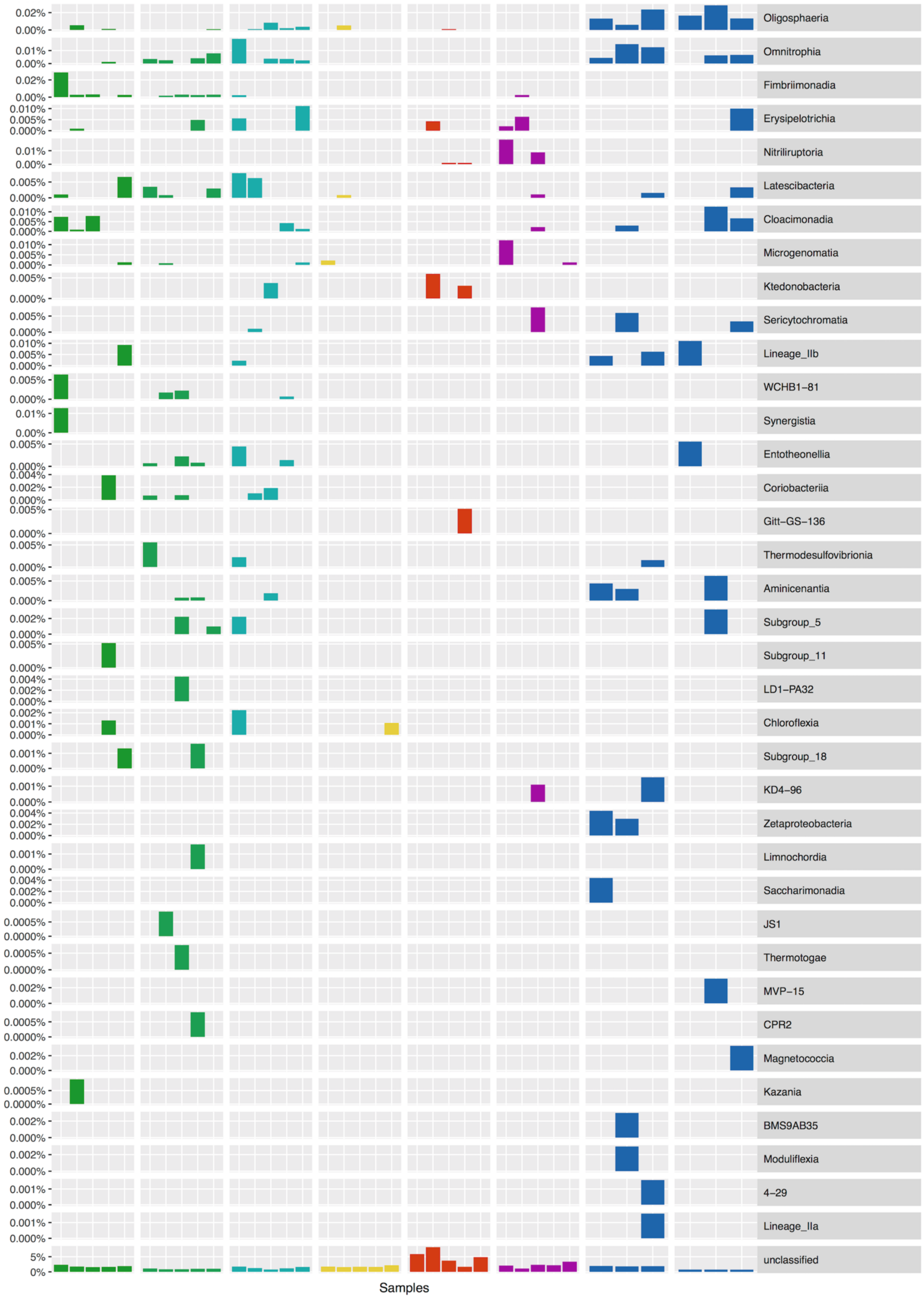
Oligosphaeria
Omnitrophia
Fimbriimonadia
Erysipelotrichia
Nitriliruptoria
Latescibacteria
Cloacimonadia
Microgenomatia
Ktedonobacteria
Sericytochromatia
Lineage_IIb
WCHB1–81
Synergistia
Entotheonellia
Coriobacteriia
Gitt–GS–136
Thermodesulfovibrionia
Aminicenantia
Subgroup_5
Subgroup_11
LD1–PA32
Chloroflexia
Subgroup_18
KD4–96
Zetaproteobacteria
Limnochordia
Saccharimonadia
JS1
Thermotogae
MVP–15
CPR2
Magnetococcia
Kazania
BMS9AB35
Moduliflexia
4–29
Lineage_IIa
unclassified
Samples

Supplementary Figure 1. Relative abundance distribution of the bacterial communities at the class level on stainless steel at different temperatures and in the seawater collected before and after the experiment. OTUs with the same affiliation were pooled.

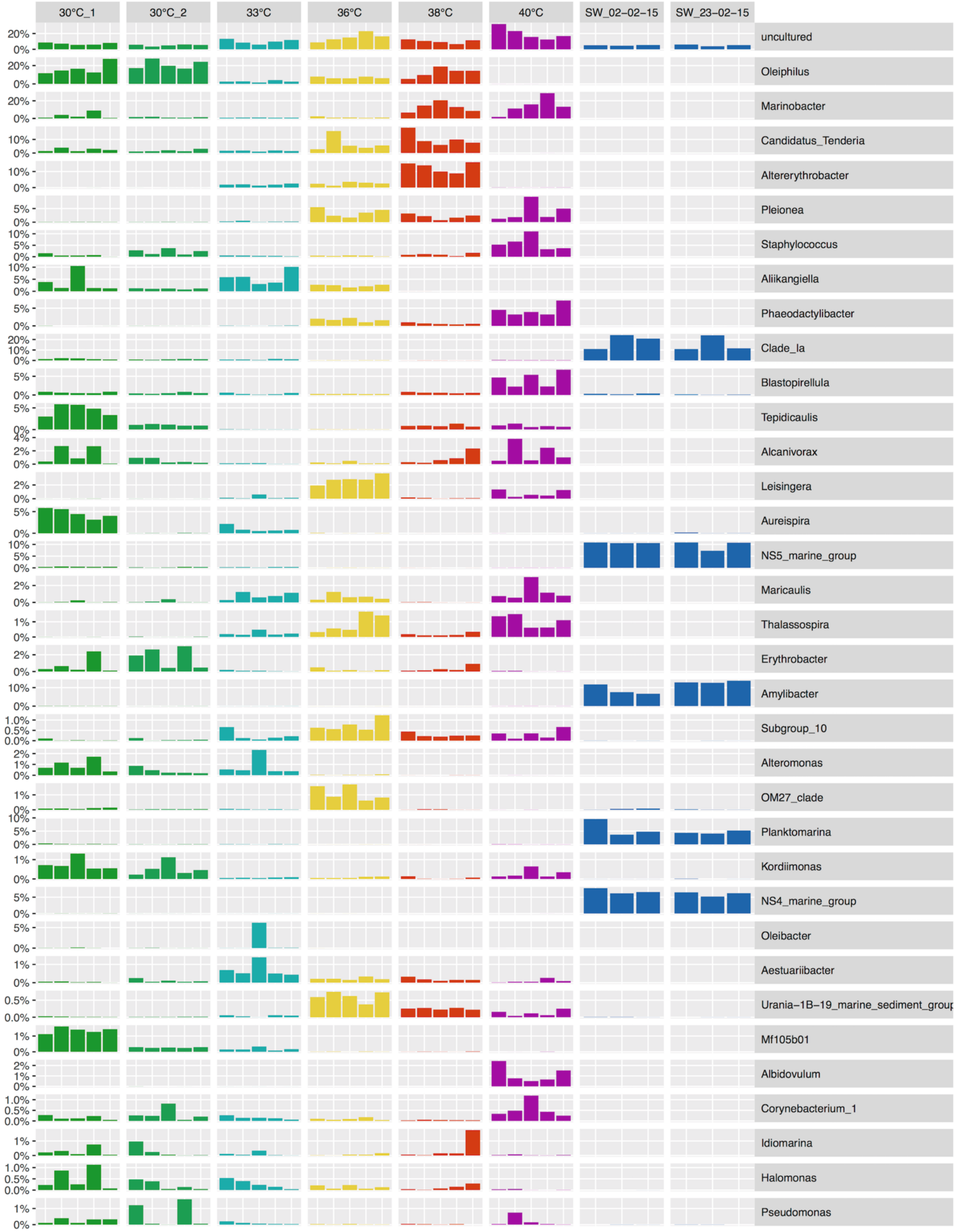

30°C_1
30°C_2
33°C
36°C
38°C
40°C
SW_02–02–15
SW_23–02–15
uncultured
Oleiphilus
Marinobacter
Candidatus_Tenderia
Altererythrobacter
Pleionea
Staphylococcus
Aliikangiella
Phaeodactylibacter
Clade_Ia
Blastopirellula
Tepidicaulis
Alcanivorax
Leisingera
Aureispira
NS5_marine_group
Maricaulis
Thalassospira
Erythrobacter
Amylibacter
Subgroup_10
Alteromonas
OM27_clade
Planktomarina
Kordiimonas
NS4_marine_group
Oleibacter
Aestuariibacter
Urania–1B–19_marine_sediment_grou
Mf105b01
Albidovulum
Corynebacterium_1
Idiomarina
Halomonas
Pseudomonas

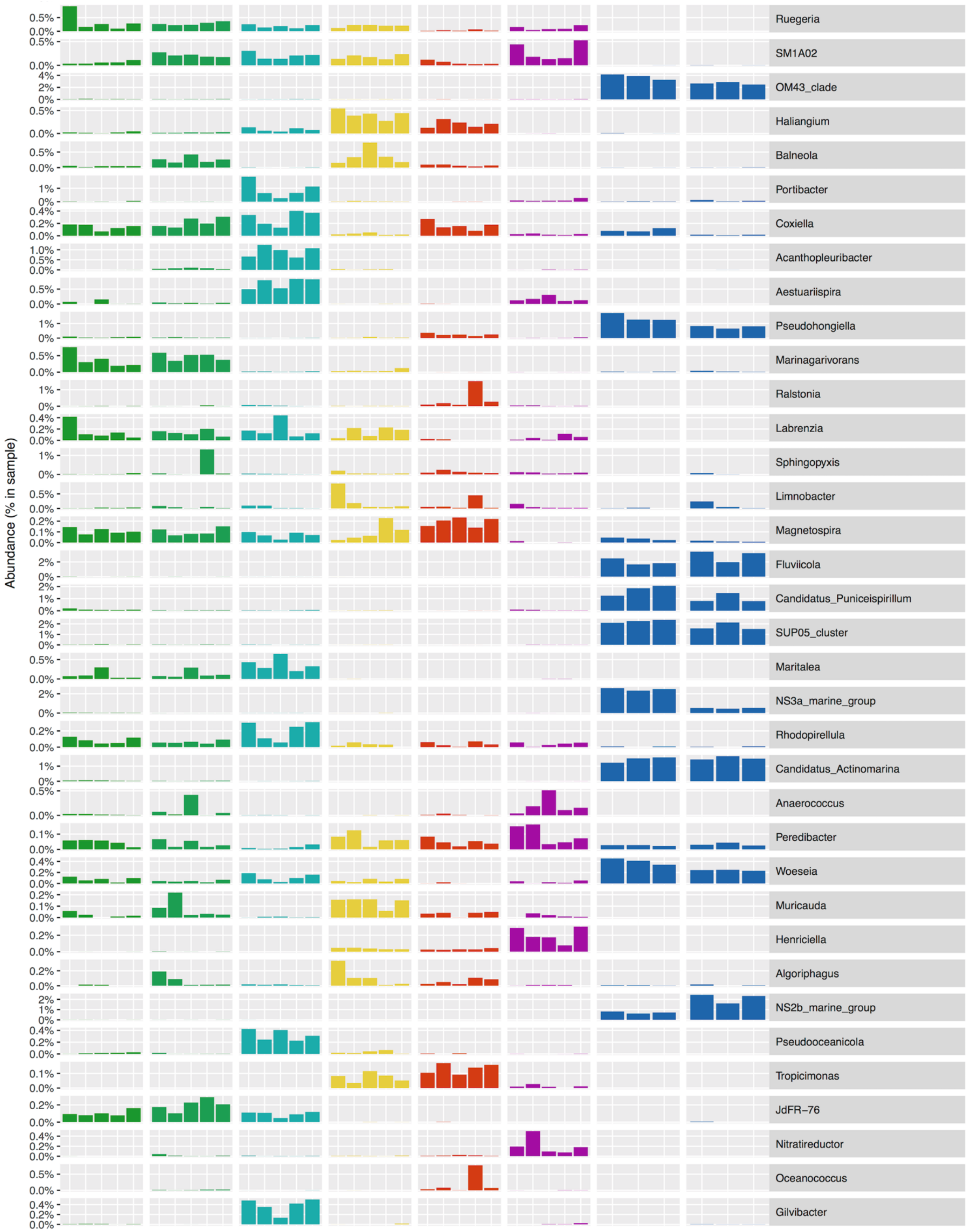
Abundance (% in sample)
Ruegeria
SM1A02
OM43_clade
Haliangium
Balneola
Portibacter
Coxiella
Acanthopleuribacter
Aestuariispira
Pseudohongiella
Marinagarivorans
Ralstonia
Labrenzia
Sphingopyxis
Limnobacter
Magnetospira
Fluviicola
Candidatus_Puniceispirillum
SUP05_cluster
Maritalea
NS3a_marine_group
Rhodopirellula
Candidatus_Actinomarina
Anaerococcus
Peredibacter
Woeseia
Muricauda
Henriciella
Algoriphagus
NS2b_marine_group
Pseudooceanicola
Tropicimonas
JdFR–76
Nitratireductor
Oceanococcus
Gilvibacter

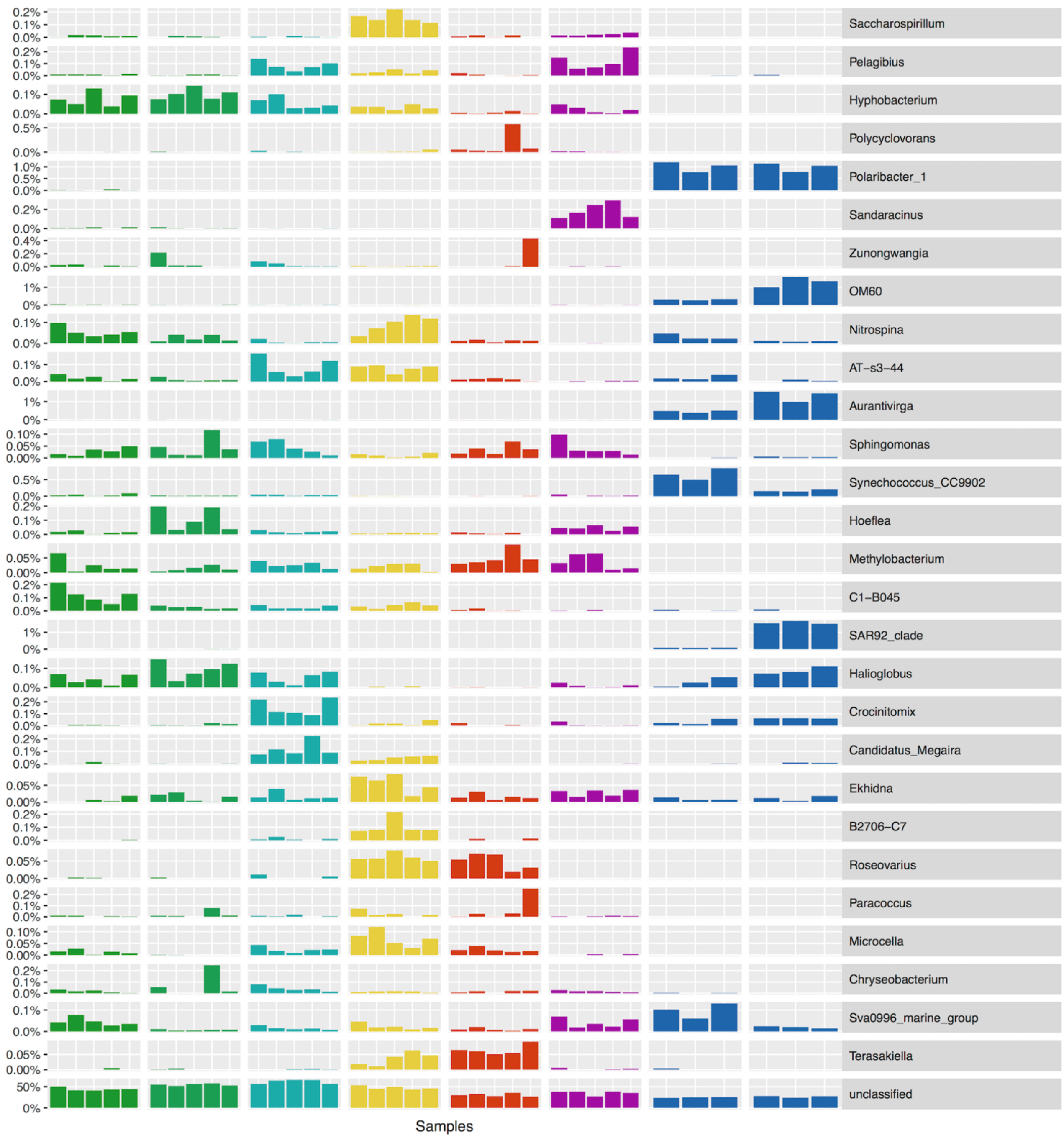

Supplementary Figure 2. Relative abundance distribution of the bacterial communities at the genus level on stainless steel at different temperatures and in the seawater collected before and after the experiment. OTUs with the same affiliation were pooled and only the 100 most abundant are shown.

36°C

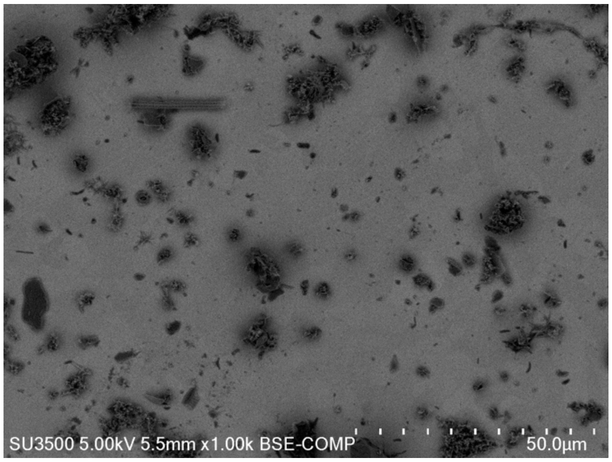
SU3500 5.00kV 5.5mm x1.00k BSE-COMP
50.0µm

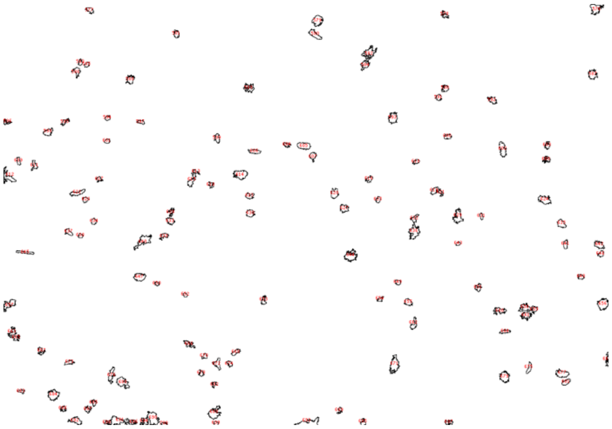

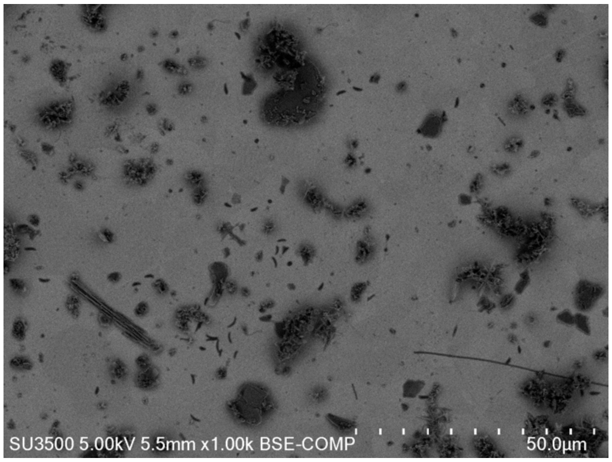
SU3500 5.00kV 5.5mm x1.00k BSE-COMP
50.0µm

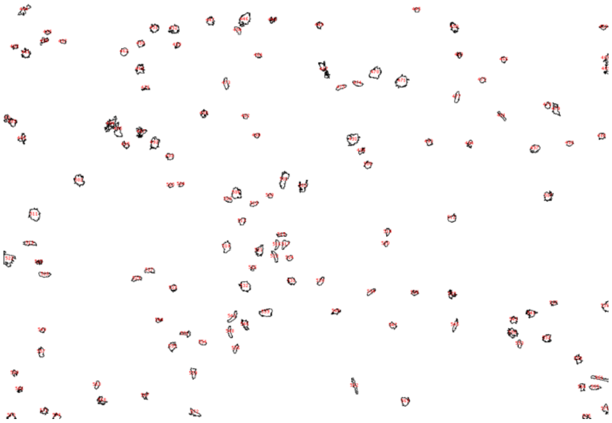

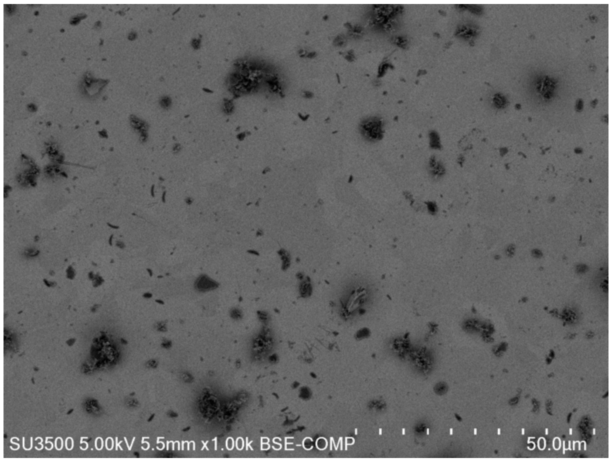
SU3500 5.00kV 5.5mm x1.00k BSE-COMP
50.0µm

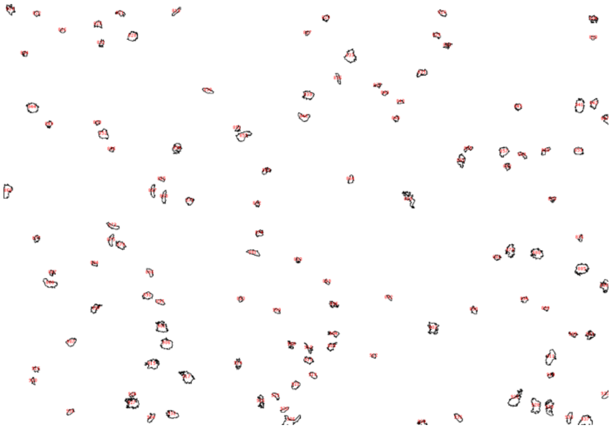

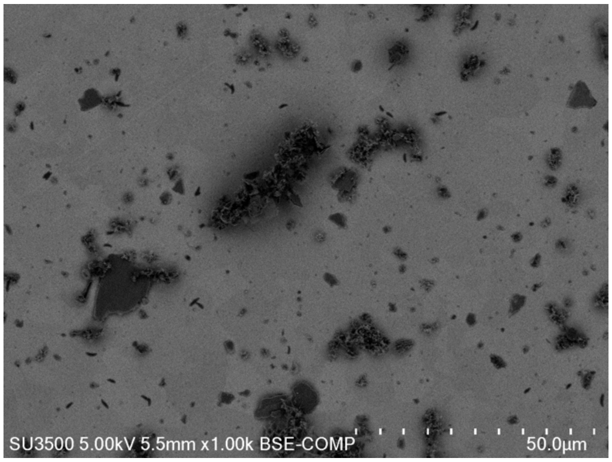
SU3500 5.00kV 5.5mm x1.00k BSE-COMP
50.0µm

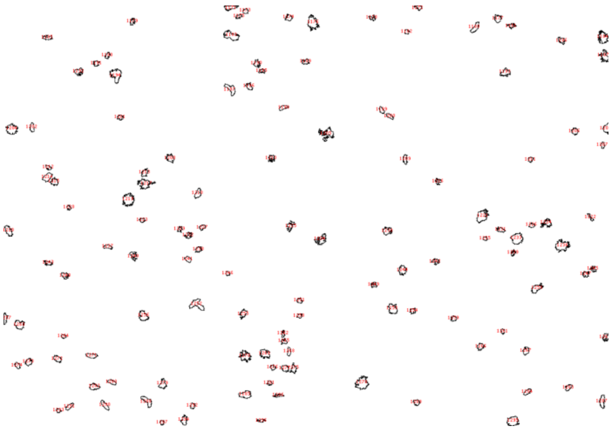

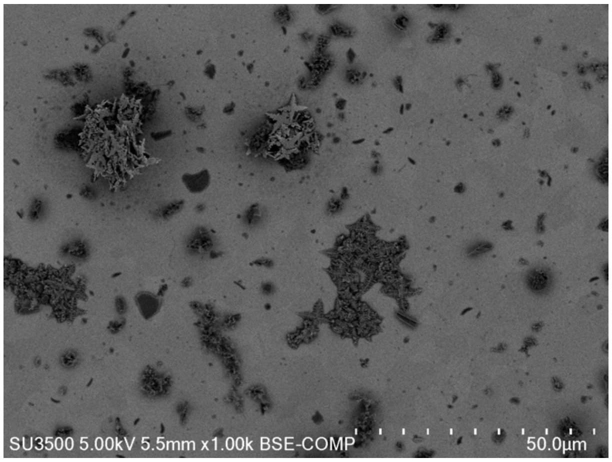
SU3500 5.00kV 5.5mm x1.00k BSE-COMP
50.0µm

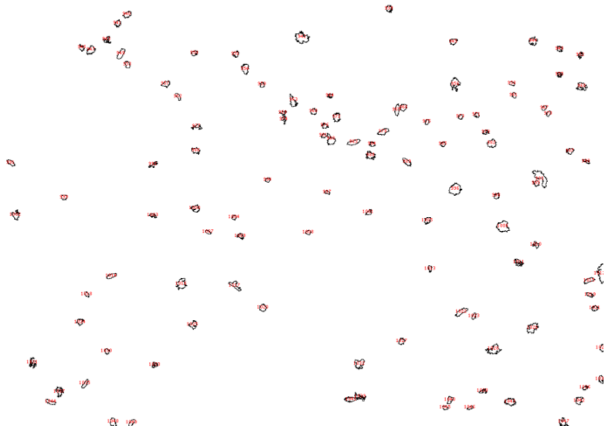

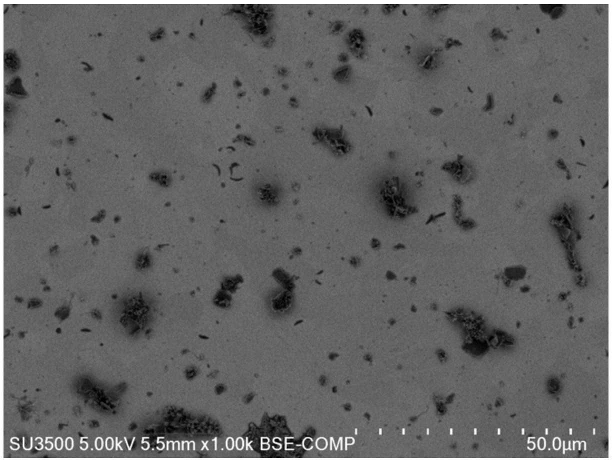
SU3500 5.00kV 5.5mm x1.00k BSE-COMP
50.0µm

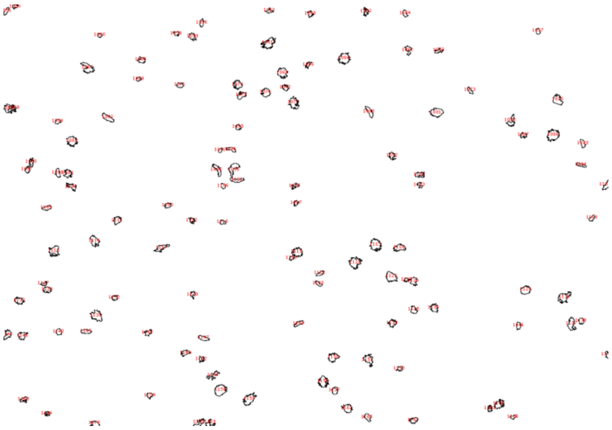

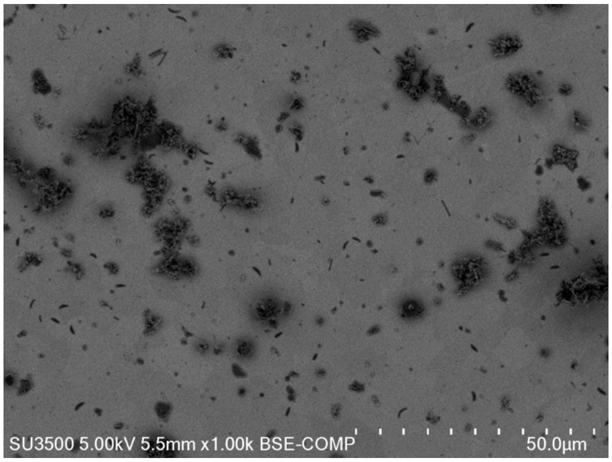SU3500 5.00kV 5.5mm x1.00k BSE-COMP<br>50.0μm	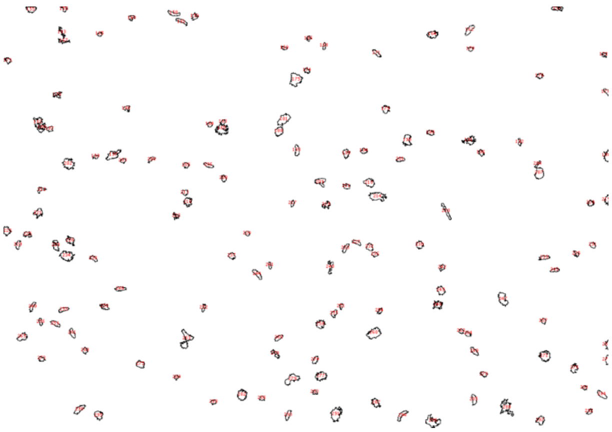
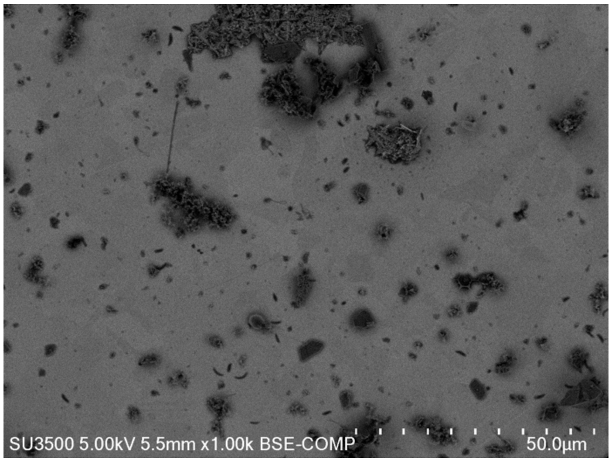SU3500 5.00kV 5.5mm x1.00k BSE-COMP<br>50.0μm	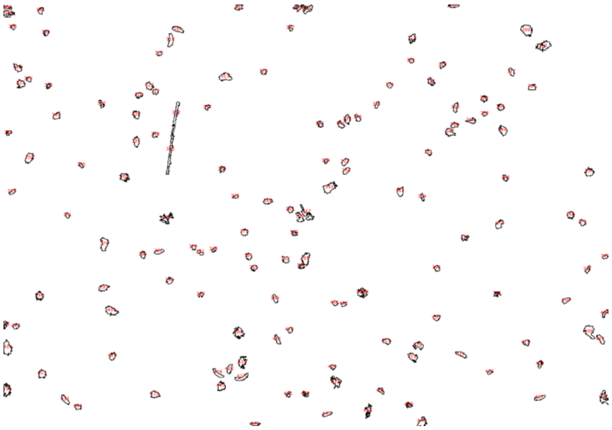
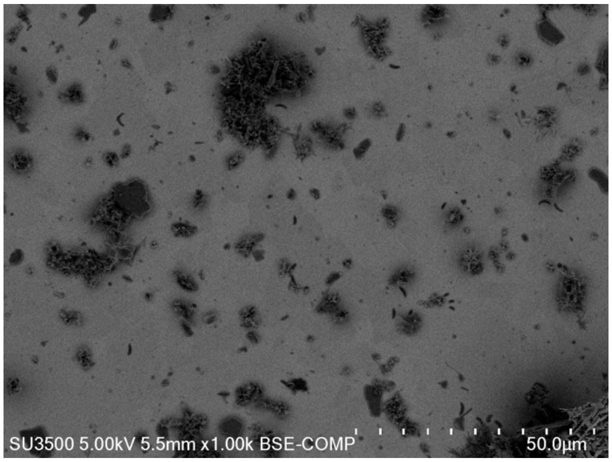SU3500 5.00kV 5.5mm x1.00k BSE-COMP<br>50.0μm	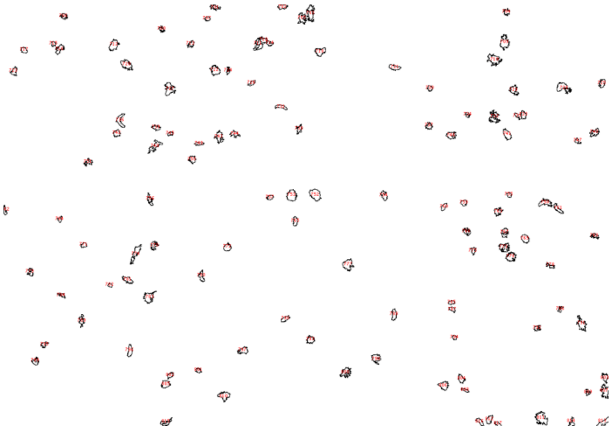

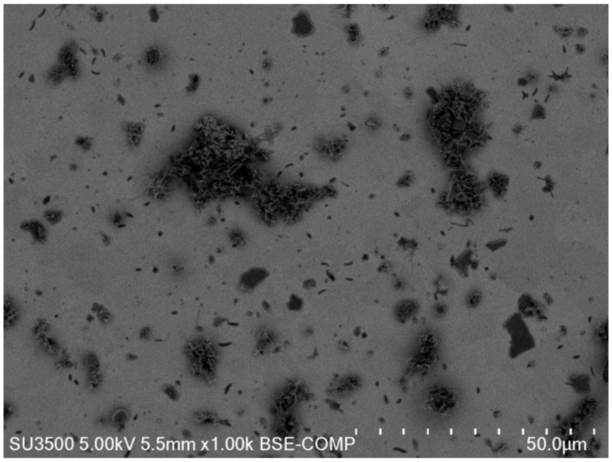
SU3500 5.00kV 5.5mm x1.00k BSE-COMP
50.0μm

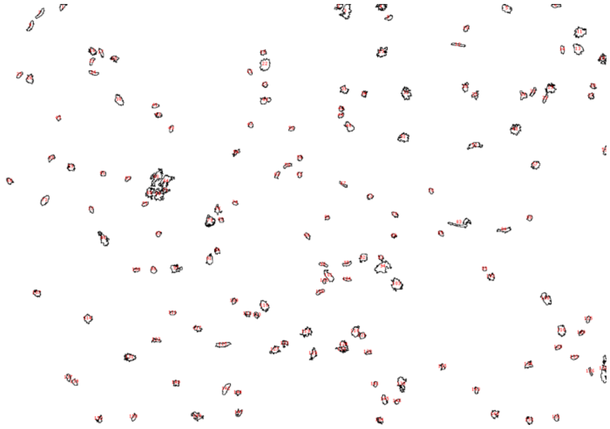

40°C

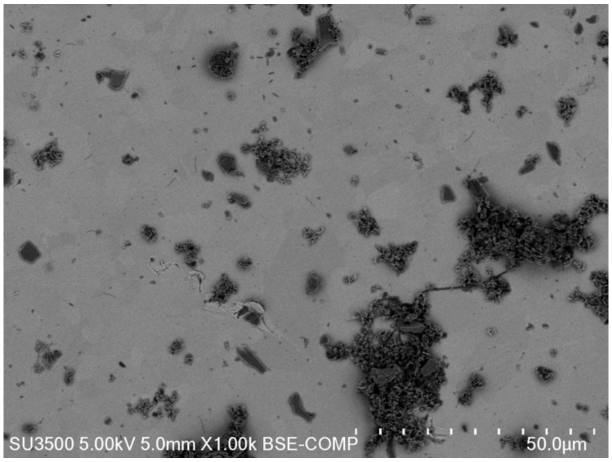
SU3500 5.00kV 5.0mm X1.00k BSE-COMP
50.0μm

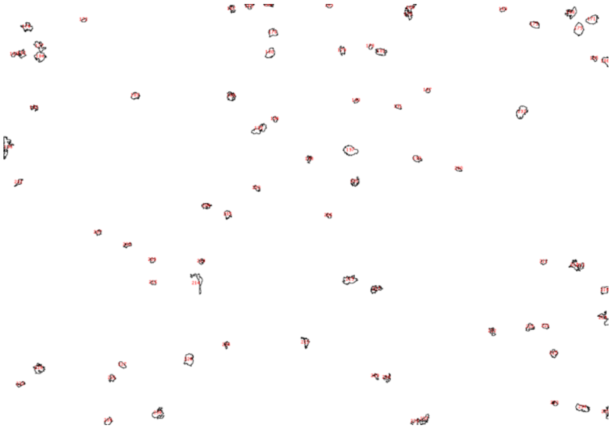

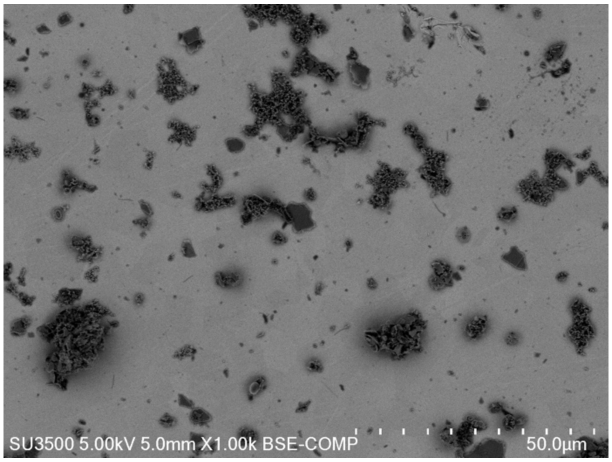
SU3500 5.00kV 5.0mm X1.00k BSE-COMP
50.0μm

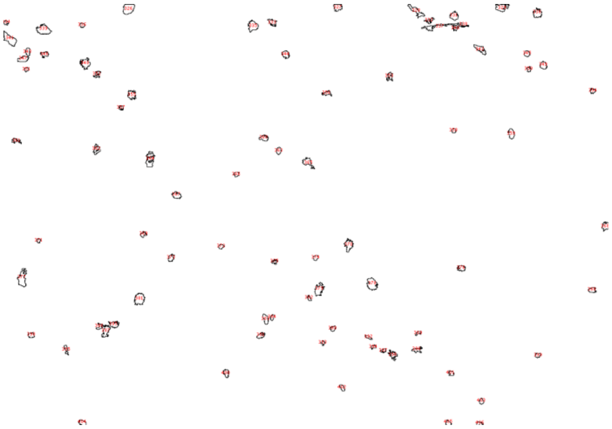

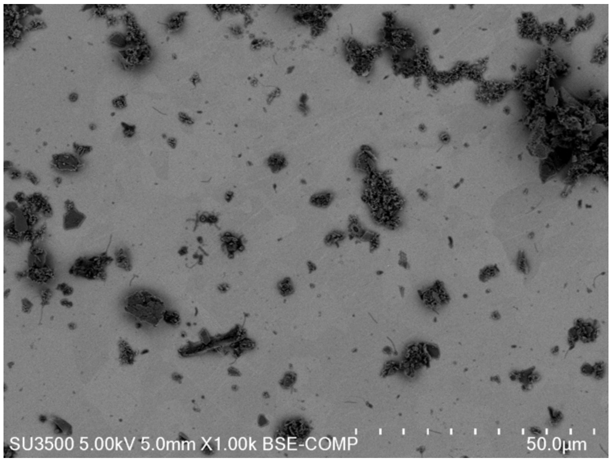<br>SU3500 5.00kV 5.0mm X1.00k BSE-COMP<br>50.0µm	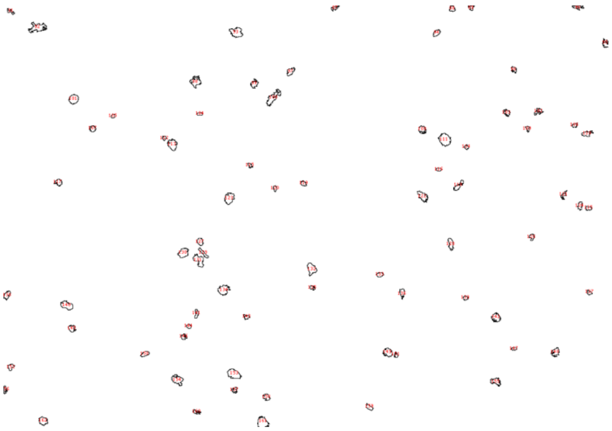
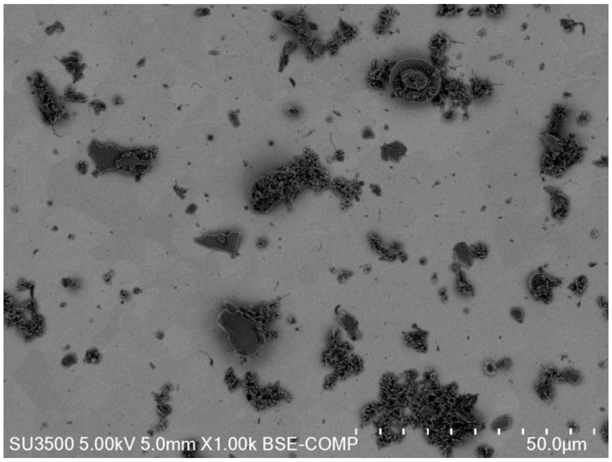<br>SU3500 5.00kV 5.0mm X1.00k BSE-COMP<br>50.0µm	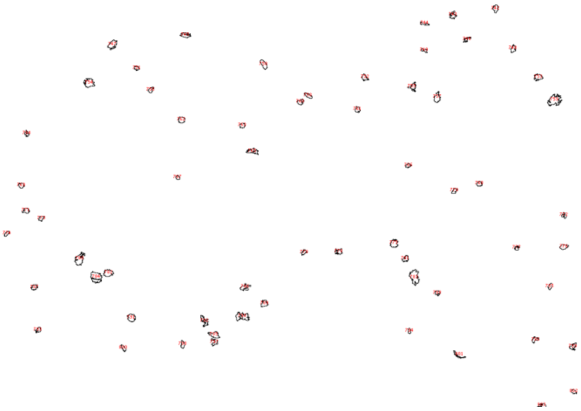
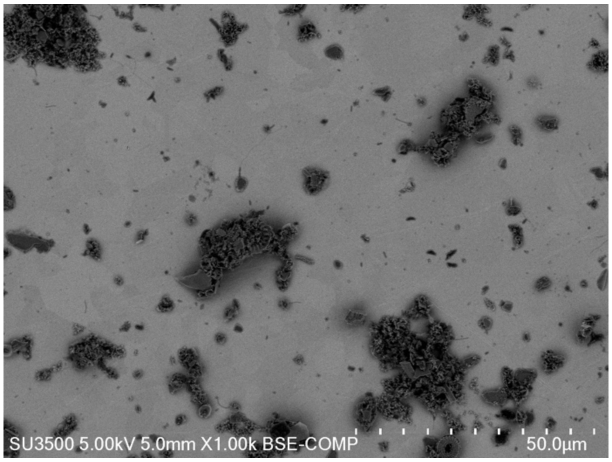<br>SU3500 5.00kV 5.0mm X1.00k BSE-COMP<br>50.0µm	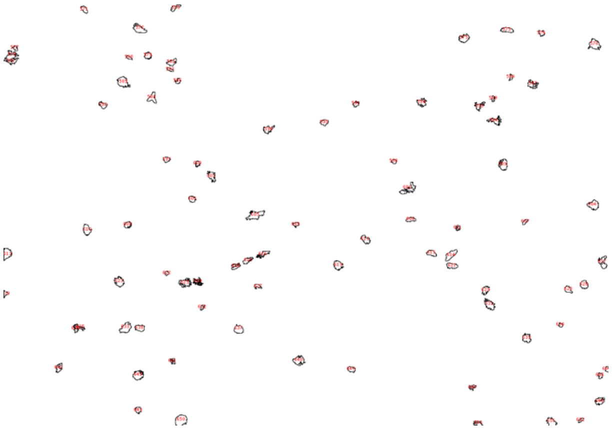

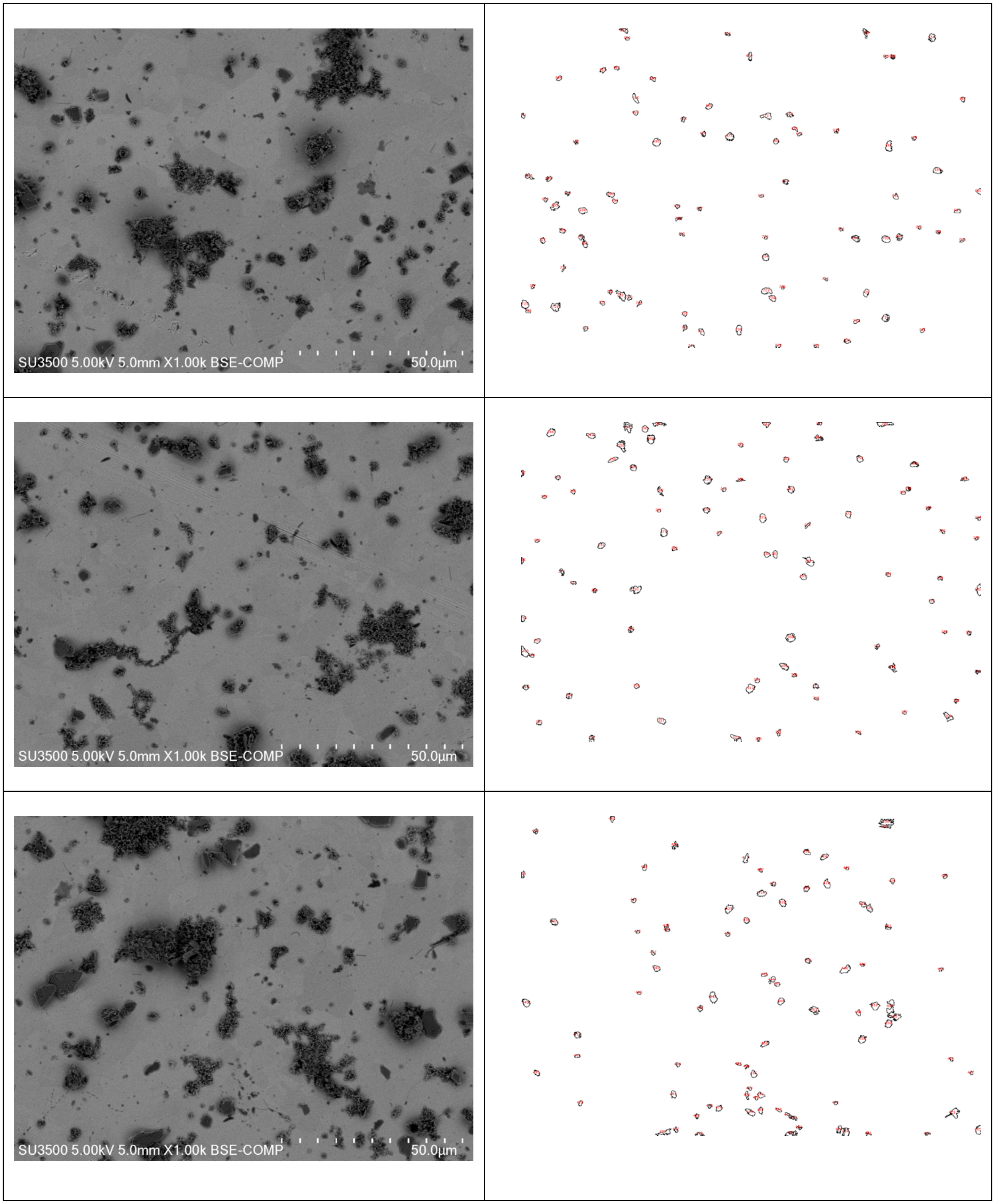
SU3500 5.00kV 5.0mm X1.00k BSE-COMP
50.0μm
SU3500 5.00kV 5.0mm X1.00k BSE-COMP
50.0μm
SU3500 5.00kV 5.0mm X1.00k BSE-COMP
50.0μm

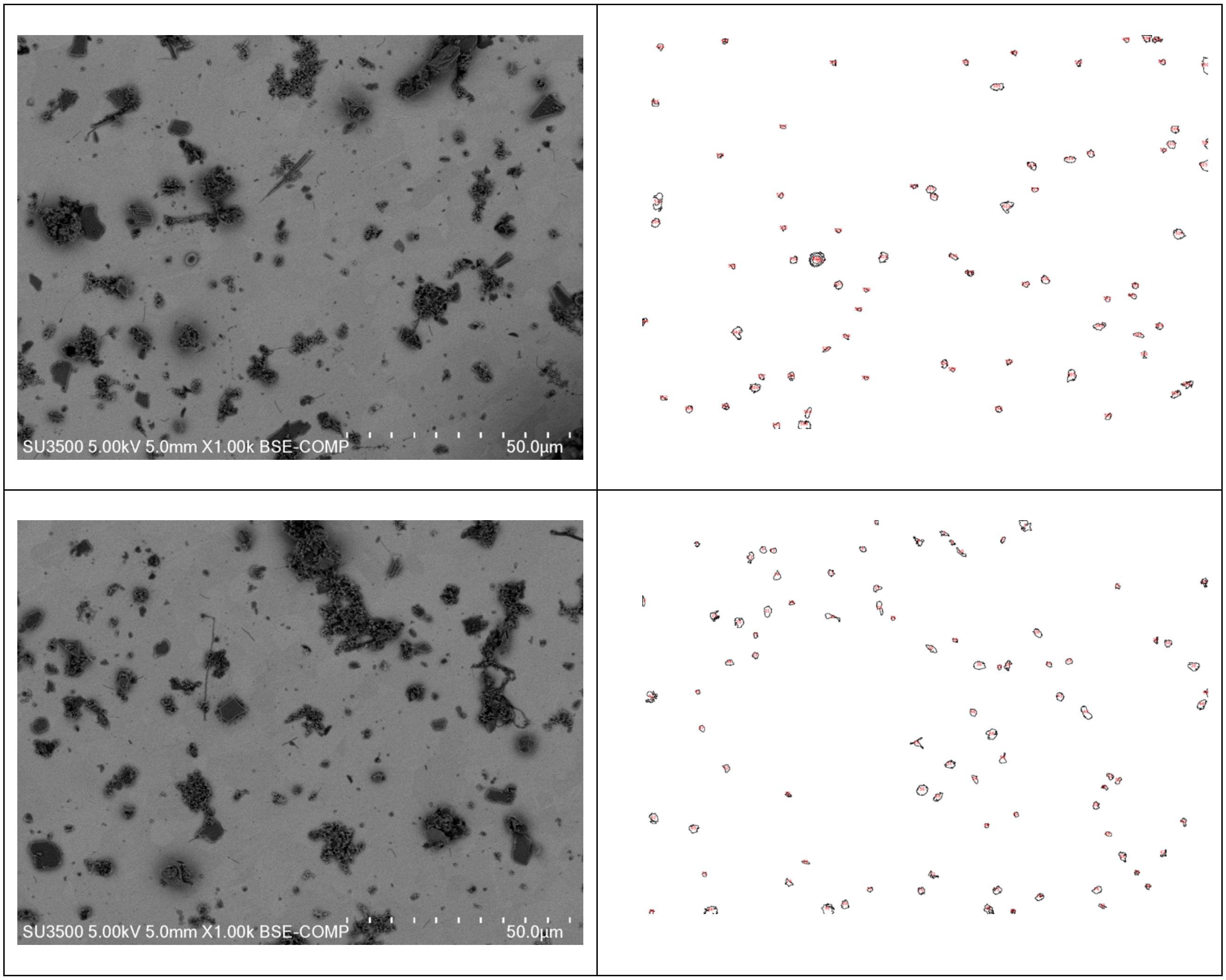

Supplementary Figure 3. SEM backscattered-electron imaging at 1K magnification for cell detection using the ImageJ software. SEM picture (left), automatic cell detection (right). After the background removal, images were converted into binary black and white with the default threshold of the software. The particle analysis was used with a minimum area of 0.5 µm2 up to 4 µm2 for cell detections.
